# l-Carnitine counteracts in vitro fructose-induced hepatic steatosis through targeting oxidative stress markers

**DOI:** 10.1007/s40618-019-01134-2

**Published:** 2019-11-08

**Authors:** A. Montesano, P. Senesi, F. Vacante, G. Mollica, S. Benedini, M. Mariotti, L. Luzi, I. Terruzzi

**Affiliations:** 1 Department of Biomedical Sciences for Health, University of Milan, Milan, Italy; 2 Metabolism Research Center, IRCCS Policlinico San Donato, San Donato Milanese, Milan, Italy; 3 IRCCS Istituto Ortopedico Galeazzi, Milan, Italy; 4 Department of Biomedical, Surgical and Dental Sciences, University of Milan, Milan, Italy

**Keywords:** Hepatic steatosis, Metabolic disease, Fructose, l-Carnitine, Lipid deposition, Oxidative stress

## Abstract

**Purpose:**

Nonalcoholic fatty liver disease (NAFLD) is defined by excessive lipid accumulation in the liver and involves an ample spectrum of liver diseases, ranging from simple uncomplicated steatosis to cirrhosis and hepatocellular carcinoma. Accumulating evidence demonstrates that high fructose intake enhances NAFLD development and progression promoting inhibition of mitochondrial β-oxidation of long-chain fatty acids and oxidative damages. l-Carnitine (LC), involved in β-oxidation, has been used to reduce obesity caused by high-fat diet, which is beneficial to ameliorating fatty liver diseases. Moreover, in the recent years, various studies have established LC anti-oxidative proprieties. The objective of this study was to elucidate primarily the underlying anti-oxidative mechanisms of LC in an in vitro model of fructose-induced liver steatosis.

**Methods:**

Human hepatoma HepG2 cells were maintained in medium supplemented with LC (5 mM LC) with or without 5 mM fructose (F) for 48 h and 72 h. In control cells, LC or F was not added to medium. Fat deposition, anti-oxidative, and mitochondrial homeostasis were investigated.

**Results:**

LC supplementation decreased the intracellular lipid deposition enhancing AMPK activation. However, compound C (AMPK inhibitor-10 μM), significantly abolished LC benefits in F condition. Moreover, LC, increasing PGC1 α expression, ameliorates mitochondrial damage-F induced. Above all, LC reduced ROS production and simultaneously increased protein content of antioxidant factors, SOD2 and Nrf2.

**Conclusion:**

Our data seemed to show that LC attenuate fructose-mediated lipid accumulation through AMPK activation. Moreover, LC counteracts mitochondrial damages and reactive oxygen species production restoring antioxidant cellular machine. These findings provide new insights into LC role as an AMPK activator and anti-oxidative molecule in NAFLD.

## Introduction

Nonalcoholic fatty liver disease (NAFLD), one of the most common cause of chronic liver diseases, is characterized by the abundant accumulation of triglycerides in hepatocytes, a condition that starts from a simple steatosis and may further progress to steatohepatitis (NASH), cirrhosis, and hepatocellular carcinoma [1, 2]. Since NAFLD patients are often overweight, over-nutrition achieves a fundamental role during the pathogenesis of hepatic lipid accumulation [3]. Several investigations have demonstrated that a fructose (F) overconsumption is involved in NAFLD progression, stimulating de novo lipogenesis, production and secretion of triglyceride and very low-density lipoprotein, and blocking fatty acid oxidation [4]. Moreover, recent data suggest how a chronically high fructose intake could inhibit AMP-activated protein kinase (AMPK), the main energy sensor of cellular metabolism, whereas its activation counteracts NAFLD progression [5]. Additionally, literature documents have indicated that fructose-rich diet is associated with oxidative stress and, in particular, with the decrease of mitochondrial biogenesis and antioxidant machine [6]. In particular, high fructose intake leads to a dysregulation of nuclear factor E2-related factor 2 (Nrf2) expression that regulates mitochondrial antioxidant function enhancing synthesis of detoxifying enzymes [7]. Recently, Sharma et al. have revealed how in mice fed high fat plus fructose, Nrf2 pharmacological activation ameliorates insulin resistance and alleviates NASH and liver fibrosis, principally decreasing oxidative stress and inflammatory [8].

If an imbalance diet and altered cellular metabolism are the main causes of NAFLD progression, eating habit modifications and, in general, weight reduction remain a first-line strategy in NAFLD management [9]. The nutritional recommendations are even more important considering that a specific pharmacological intervention for NAFLD has not yet been identified. In effect, the various pharmacological treatments use specific drugs for coexisting diseases, namely the glucagon-like peptide 1 and the cotransport antagonist 2 sodium/glucose (SGLT-2) for the control of glycaemia, in association with vitamin E supplementation [10]. Recently, novel therapeutic options for NAFLD have been proposed including activation of farnesoid X receptor (FXR) that ameliorates fibrotic and inflammatory damages [11, 12]. However, Mediterranean diet prefers low glycemic index products and antioxidant foods and, in general, weight reduction extremely effectively counteracts NAFLD progression [9].

In details, diet supplements or nutraceutical agents having cellular antioxidant activity are likely to have therapeutic capacities in NAFLD [9, 13]. For example, in rat fed with high fructose diet, curcumin relieves NAFLD activating Nrf2 signaling [14], while vitamin E significantly reduces the overproduction of ROS induced by fructose [6]. In particular, various clinical trials are underway to demonstrate the effectiveness of vitamin E in NAFLD management: Vilar-Gomez et al. have demonstrated that vitamin E supplementation improved clinical outcomes in diabetic and no diabetic patients with NASH ameliorating fibrosis or cirrhosis [15]. Moreover, Sanyal et al. have compared vitamin E and pioglitazone efficacy in liver steatosis observing that vitamin E had a greater effectiveness for the treatment of nonalcoholic steatohepatitis in adults without diabetes [16].

l-Carnitine (LC) plays a critical role in a number of intracellular and metabolic functions, such as fatty acid transport into the mitochondria, stabilization of cell membranes, and reduction of serum lipid levels [17]. Moreover, recent evidence has showed, as LC is also a potent antioxidant: in vitro studies, performing mouse myoblasts [18], rat cardiomyocytes [19] and human osteoblastic cells [20], LC supplementation decrease ROS overproduction and cellular antioxidant defense system.

Based on LC proprieties, Malaguarnera et al. studied how oral LC supplementation improved liver functions and histological patterns in patients with NASH [21]. However, LC mechanism of its protective effect on NAFLD remains to be elucidated and LC action on hepatic damages induced by high fructose intake should be investigated.

The objective of the present study was to clarify the underlying mechanisms of LC action in counteracting fructose-induced hepatic steatosis using human hepatocytes (HepG2 cells). In details, we investigated how LC could regulate fat deposition and mitochondrial anti-oxidative processes.

## Materials and methods

### Materials

All utilized reagents were purchased from Sigma Chemical Co. (St. Louis, MO, U.S.A.).

Primary antibodies against calnexin (H-70),GAPDH (FL-335), AMPKα1/2 (H-300), pCaMKII (22B1), Nrf2 (C-20), PGC1 α (H-300), SOD2 (FL-222), peroxidase-conjugated secondary antibodies for western blot analysis and rhodamine/FITC-conjugated antibodies for immunofluorescence studies were purchased from Santa Cruz Biotechnology (Santa Cruz-CA, U.S.A.). Primary antibody p-AMPKα1/2 (Thr 172) and CaMKII (6G9) was purchased from Cell Signaling Technology (Danvers-MA, U.S.A.).

Compound C (dorsomorphin), AMPK inhibitor, was acquired from Aurogene (Roma, Italy).

### Cell line, culture conditions

**Fig. 1:**
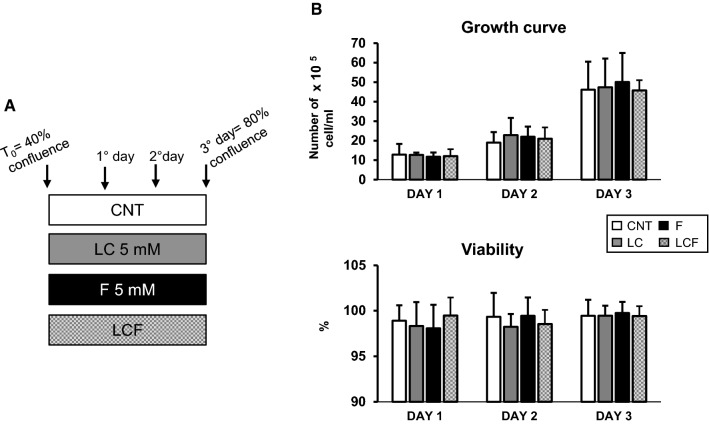
Treatment action on proliferative phase of HepG2 cells. **a** Experimental scheme of HepG2 treatments. **b** Growth curve and viability determination: treatment with LC, F or LCF not altered HepG2 proliferative potential. Viability graph showed the absence of cell mortality in all treatment conditions. For growth curve and viability, a two-way ANOVA test followed by Tukey’s multiple comparison test was performed

The human hepatocellular carcinoma cell line HepG2 was obtained from the European Collection of Cell Cultures (ECACC) and maintained in MEM containing 10% fetal bovine serum (FBS), 1% penicillin streptomycin, 1% glutamine, and 1% MEM non-essential amino acid. The cells were incubated in a humidified atmosphere of 5% CO_2_ at 37 °C and passaged by trypsinization when they reached 80% confluence. The culture medium was changed every day, following literature indications. For all experiments, HepG2 were treated with 5 mM fructose (F) and 5 mM l-Carnitine (LC) (single or combined treatment), as indicated in the treatment plan in Fig. 1a. These doses were chosen based on literature data [18, 19]. We studied HepG2 growth capacity to evaluate the absence of cytotoxic effects. Growth curve and cell viability test were performed as previously described. Briefly, HepG2 cells (2 × 10^5^) were plated at 40% confluence and grown in MEM. The cells were treated or not with F and LC for 1, 2 or 3 days. Every day cells were trypsinized, stained with trypan blue, and counted using hemocytometer. Cell viability was calculated by dividing the no stained viable cell count by the total cell count [22]. To abolish AMPK activity, we added AMPK inhibitor, compound C, at 10 μM for 72 h [23].

### Western blot analysis

HepG2 cells were grown in 100 mm culture dishes with or without LC combined or not with F. Cellular extracts were obtained lysing the cells in RIPA buffer as previously described [24]. Thirty micrograms of proteins were separated by SDS–polyacrylamide gel electrophoreses (SDS-PAGE) and electrophoretically transferred to nitrocellulose membranes (Potran^®^, Whatman^®^ Schleicher & Schuell). The blots were then blocked and probed with specific primary antibodies, followed by incubation with anti-species-specific secondary antibodies.

To confirm equal protein loading per sample, we used antibody anti-calnexin or anti-GAPDH. Detection of specific proteins was performed by enhanced chemioluminescence reagent (Western Lightning ECL Pro, Perkin Elmer). Quantitative measurement of immunoreactive band intensities was performed by densitometry analysis using the Scion Image software (Scion Corporation, Frederick, MD, USA). Only for AMPK inhibitor experiments and CaMKII, immunoreactive bands were visualized by Uvitec Alliance LD9 gel imaging system (Uvitec, Cambridge, UK).

Data were then presented as ratio between treated cells and control.

### Immunofluorescence

HepG2 cells were grown on coverslips with or without 5 mM LC combined or not with 5 mM F. After 72 h of treatment, cells were washed with PBS, fixed in 4% paraformaldehyde and lastly, rinsed three times in PBS. Then, the cells on the coverslips were incubated for 30 min at room temperature with 1% bovine serum albumin in PBS with 0.2% Triton X-100. HepG2 cells on coverslips were then immunostained with specific primary and secondary antibodies, rhodamine- or FITC-conjugated, and nuclei were revealed with DAPI staining.

The MITO CytoPainter mitochondrial indicator (Prodotti Gianni, Italy) is a hydrophobic compound that easily permeates intact live cells remaining trapped in mitochondria. This staining was performed on HepG2 live cells.

Cell ROX^®^ oxidative stress reagents (Thermo Fisher Scientific, Italy) are fluorogenic probes designed to reliably measure reactive oxygen species (ROS) in live cells. The cell-permeable reagents are non-fluorescent or very weak fluorescent, while in a reduced state and upon oxidation exhibit strong fluorogenic signal. Cell ROX^®^ Orange Reagents are localized in the cytoplasm. ROS production by mitochondria was visualized in fluorescence microscopy using the MitoSOX™ red reagent (Thermo Fisher Scientific, Italy). These staining were performed on HepG2 lived cells after 72 h of treatment.

Slides were mounted with Moviol. Cells were observed using Nikon Eclipse 50I microscopy and images were captured using Nis-Elements D 4.00 software (Nikon Instruments Europe BV-Netherlands).

### Oil Red O Staining

Oil Red O bases this technique on the staining of intracellular lipid droplets.

After treatments with LC and F, HepG2 cells were washed with PBS and fixed using 4% formalin for 30 min. Subsequently, the cells were washed twice-using ddH_2_O and incubated with Oil Red O solution for 15 min at RT. To remove background staining, the cells were washed three times with a 60% isopropanol solution for 5 min. For quantitative analysis of Oil Red O contents levels, isopropanol was added to each samples and then shaken at room temperature for 5 min. The absorbance was read at 490 nm with Bio-rad iMark Microplate Reader (Bio-Rad Laboratories, Inc., Hercules, CA, USA).

### Statistical analysis

All experiments were performed three times. All data are presented as mean ± SD.

Shapiro–Wilk test was used to ascertain normality of the data distribution.

For variables with Gaussian distribution, one-way analysis of variance (ANOVA) was used to compare means among the groups (followed by Tukey’s post hoc test), whereas for variables with non-Gaussian distribution, Kruskal–Wallis test was used to compare means among the groups (followed by Dunn’s post hoc test). Results were considered significant when *p* ≤ 0.05.

Statistical analysis was performed with specific statistical packages (Prism v 7.00 GraphPad Software, San Diego, CA, USA).

## Results

In this study, the model of steatosis was mimicked using human hepatocytes HepG2 treated with 5 mM F for 72 h (Fig. 1a). We first verified that 5 mM F, 5 mM LC and combination of F and LC did not cause cytotoxic effects. As shown in Fig. 1b, these treatments did not modify growth curve and HepG2 vitality. We, therefore, investigated F, LC and LCF action on lipid accumulation.

### HepG2 intracellular lipid deposition

**Fig. 2:**
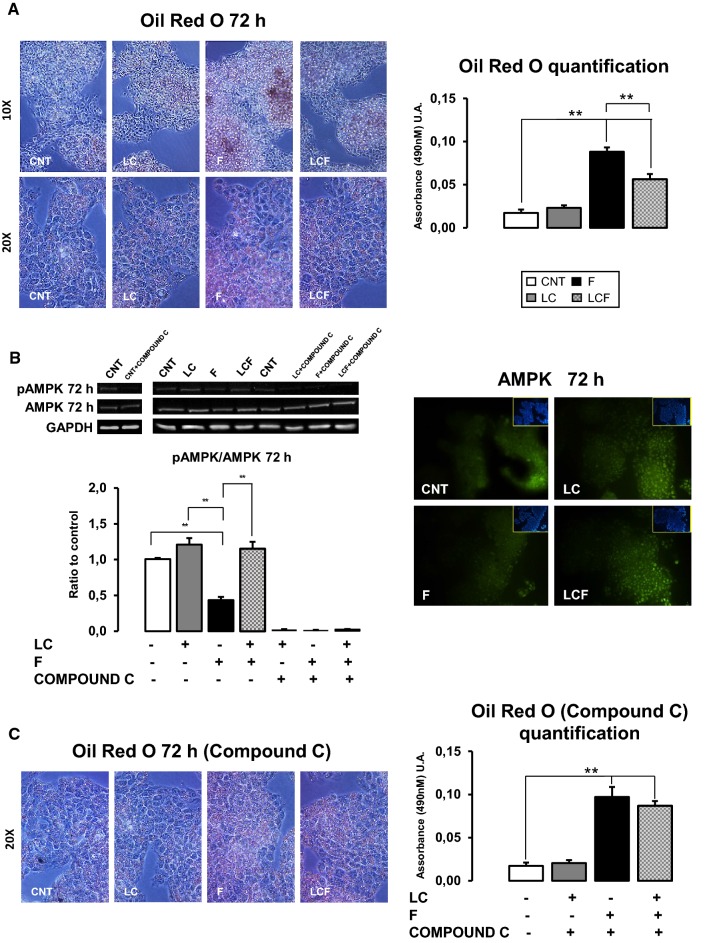
l-Carnitine role on intracellular lipids deposition. **a** Oil Red O coloration at 72 h of treatment showed how LC does not change intracellular fat deposition in respect to CNT. F treated HepG2 showed a higher vesicular lipid deposition compared to CNT, while LC addition diminished lipid accumulation. The stained lipid content was quantified by measuring absorbance at 490 nm. Objective 10X–20X. **b** Western blot analysis reported LC positive action on AMPK protein activation at 72 h of treatment: LC counteracted F action that significantly decreased AMPK activity. LC action was abolished by 10 μM Compound C (an AMPK inhibitor). Immunofluorescence assay showed the increase of AMPK protein expression in HepG2 cells treated with LC alone or in combined condition LCF with respect to CNT and F at 72 h of treatment. Objective: 20X. **c** Lipid deposition in F and LCF condition, after treatment with 10 μM Compound C (an AMPK inhibitor), was superimposable (Fig. 2c). Moreover, in CNT and LC, Compound C did not modify minimal lipid accumulation observed. The stained lipid content was quantified by measuring absorbance at 490 nm. ANOVA-Tukey post test: **p ≤ 0.01. Representative immunoblots, obtained using Uvitec Alliance LD9 gel imaging system, are shown

After 72 h, in CNT condition, a minimal lipid deposition occurred, as expected [5], and HepG2 cells treated with LC displayed a level of lipid superimposable to CNT (Fig. 2a). F-treated HepG2 showed a higher vesicular lipid deposition compared to CNT, while LC addition blunted lipid accumulation, as revealed by Oil Red quantification (Fig. 2a).

### l-Carnitine effects on AMPK

We hypothesized that lipid droplets increase was linked to AMPK activity.

Figure 2b shows that F significantly decreased AMPK activity, while LC supplementation counteracted this alteration. Above all, when HepG2 were treated with 10 μM compound C (an AMPK inhibitor), lipid deposition in F and LCF condition was superimposable (Fig. 2c). Moreover, in CNT and LC, compound C did not modify minimal lipid accumulation observed. At the same time, we observed that AMPK activity is abolished in F, LC and LCF conditions (Fig. 2b).

### l-Carnitine effects on CaMKII

**Fig. 3:**
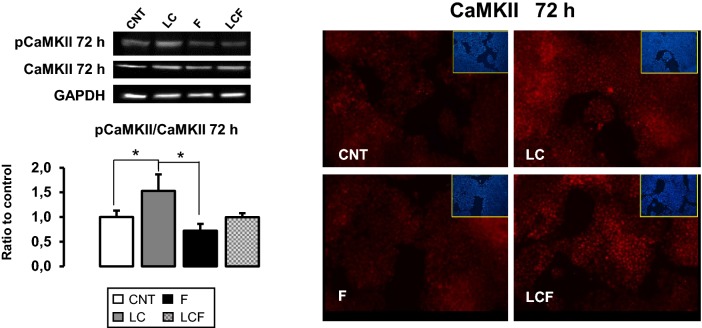
l-Carnitine role on CaMKII protein content. Immunofluorescence and western blot assay at 72 h of treatment showed how LC promoted CaMKII protein expression in HepG2 in respect to CNT and in respect to F in combined condition (LCF). Objective: 20X. Representative immunoblots, obtained using Uvitec Alliance LD9 gel imaging system, are shown

AMPK can be activated by intracellular calcium levels, in the same manner of calcium/calmodulin-dependent protein kinase II (CaMKII) [25] and our group has recently demonstrated that LC could regulate the intracellular calcium levels [26]. As shown in Fig. 3, 72 h of LC treatment stimulates CaMKII protein expression and increases CaMKII activation. In F-treated HepG2 cells, CaMKII activation is not modified compared to CNT and the same effect is observed in LCF condition.

### l-Carnitine effects on mitochondrial biogenesis and anti-oxidative response

**Fig. 4:**
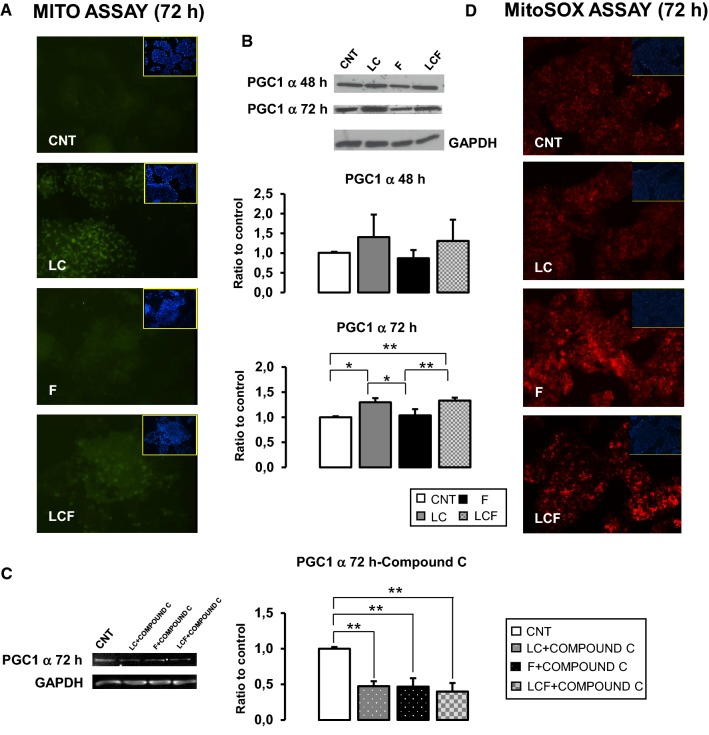
l-Carnitine role on mithocondria. **a** MITO CytoPainter staining revealed that, at 72 h of treatment, LC seems to be able to rise the signal of activated mitochondria in respect to CNT and in respect to combined condition (LCF). Objective 20X. **b** Western blot quantification reported PGC1 α increase after LC treatment. This action resulted significant after 72 h of treatment. It is important to note how this effect resulted also corroborated in the combined condition (LCF), in which LC can counteract F action. **c** Western blot analysis of PGC1 α after 72 h of treatments performed in association with AMPK inhibitor: LC positive action on PGC1 α protein content was abolish indicated that LC action is AMPK-mediated. **d** Mito SOX staining shown that, at 72 h of treatment, LC does not increase the level of mitochondrial ROS compared to CNT. In combination with F, LC appears to be able to reduce the levels of mitochondrial ROS compared to F. ANOVA-Tukey post test: *p ≤ 0.05, **p ≤ 0.01. Representative immunoblots are shown

Our group has recently demonstrated that LC could regulate cellular anti-oxidative responses [18–20, 26]. Since mitochondria are the primary intracellular regulator of ROS balance, LC effect on mitochondrial activity in HepG2 cells was studied. Figure 4a revealed that after 72 h of treatment, LC seems to be able to increase fluorescence signal linked to mitochondria in respect to CNT, while F seems decreases it. Peroxisome proliferator-activated receptor gamma coactivator 1 (PGC1 α) is a key regulator of mitochondrial dynamics and biogenesis [27]. Figure 4b shows how 72 h of LC treatment significantly increased PGC1 protein levels in respect to CNT and F: LC counteracted PGC1 α expression inhibition by F in LCF condition. Moreover, when HepG2 were treated with Compound C for 72 h, PGC1 α protein increase is completely inhibited in all conditions (Fig. 4c), showing an AMPK-dependent LC and F activity on PGC1.

These results suggested that LC, both alone and in the presence of fructose, could improve mitochondria ROS production. To verify this speculation, we evaluated, with a specific fluorescence probe (MitoSOX™), mitochondrial ROS production. As reported in Fig. 4d, in F-treated HepG2 fluorescence intensity is increased in respect to CNT and LC, while in LCF condition, ROS signal is attenuated. In HepG2 treated with LC, ROS label is similar to CNT.

**Fig. 5:**
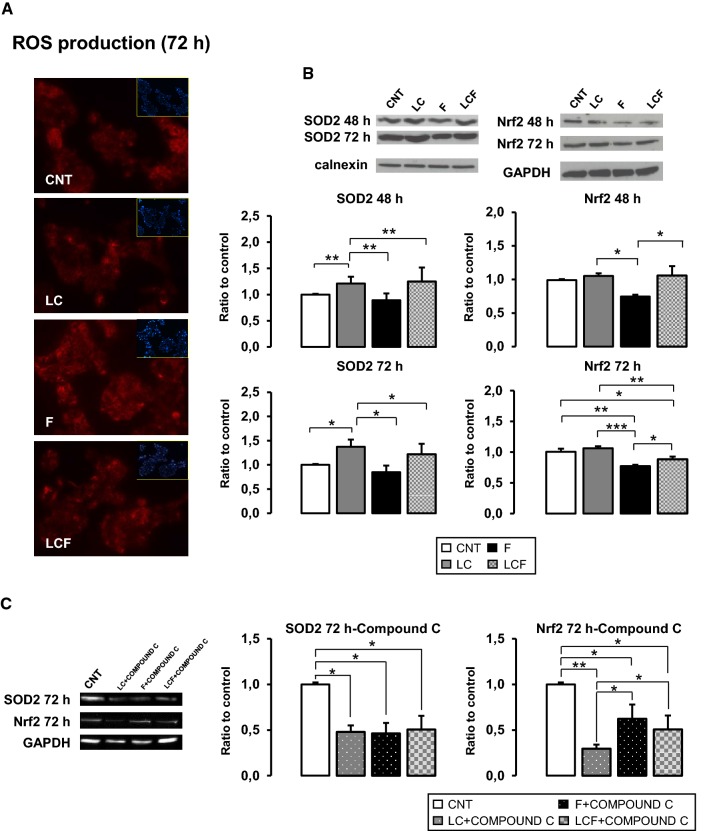
l-Carnitine role on ROS production and antioxidant factors. **a** Cell ROX assay after 30 min of H2O2 stress stimulus showed LC protective action in respect to CNT: LC treatment decreased reactive oxygen species production in HepG2 cells after 72 h of treatment. This action was confirmed also in the combined condition (LCF). Objective: 20X. **b** Western blot analysis of SOD2 and Nrf2 protein content after 48 and 72 h of treatment. LC significant promoted SOD2 protein levels in HepG2 in respect to control and in respect to F in the combined condition (LCF). In the similar manner, LC increased Nrf2 protein content after 48 h and 72 h of treatment alone and in combined condition (LCF). **c** Western blot analysis of SOD2 and Nrf2 protein content after 72 h of treatments LC, F and LC + F in association with AMPK inhibitor Compound C: SOD2 showed a minimal residual expression in all three conditions suggesting that its expression is regulated in AMPK-dependent and independent manner. Nrf2 protein content is significantly decreased in LC treated cells, while a residual expression was present in F condition suggesting that F could also promotes Nrf2 content in an AMPK-independent manner. ANOVA-Tukey post test: *p ≤ 0.05, **p ≤ 0.01, ***p ≤ 0.001. Representative immunoblots are shown

Additionally, we investigated whether LC antioxidant effect is limited to mitochondria or involved other cellular compartments. Using ROX Cell ROX^®^ Oxidative Stress Reagents, we observed that after 30 min of H_2_O_2_ stress, ROS production was reduced following 72 h of pre-treatment with LC, as shown in Fig. 5a. In details, LC seems to be able to decrease ROS response both alone (LC) and in the presence of fructose (LCF).

Finally, LC supplementation significantly increased SOD2 protein levels after 48 h and 72 h of treatment, as reported in western blot assay (Fig. 5b). Furthermore, in F condition, SOD2 protein levels were significant decreased, while LC counterbalanced F effects in HepG2 cells treated with combined stimuli (LCF).

Moreover, in Fig. 5b, western blot assay showed how LC treatment significantly increased Nrf2 protein content in respect to CNT and F stimulation.

Furthermore, Fig. 5c shows that 72 h compound C treatment significantly decreased LC positive action on SOD2 and Nrf2, indicating an AMPK-dependent modulation by LC. In details, SOD2 showed a minimal residual expression in all three conditions (LC, F, LC + F), suggesting both an AMPK-dependent and independent modulation. While, NRF2 protein increase was significantly inhibited by AMPK inhibitor in LC-treated HepG2, but it maintained a residual expression in F presence, suggesting that F could also enhance NRF2 content in an AMPK-independent manner.

## Discussion

Excessive consumption of fructose, especially sugar-sweetened beverages, is strictly associated with the development of liver steatosis [4, 5]. NAFLD is emerging as a primary health problem in the world also considering its association with diabetes and cardiovascular disease [1, 3].

Now, specifically therapies for NAFLD are limited [10–12] and currently lifestyle changes, i.e. diet modifications and physical activity enlargement, are effective in the treatment of liver steatosis [9]. However, several patients are not agreeable with healthy these indications. Consequently, finding drugs or natural compound that are more useful for NAFLD therapy has become one of the hottest research fields [13].

We investigated in fructose-induced steatosis HepG2 cells, the effects of 5 mM LC to clarify LC action in countering NAFLD.

Our data demonstrate that LC supplementation can reduce HepG2 intracellular fructose-induced lipid accumulation, recovering AMPK activity suppressed by fructose treatment (Fig. 2a). Use of an AMPK inhibitor (compound C) abolishes LC effect, confirming AMPK-mediated LC action (Fig. 2c). Recently, Woods et al. have revealed, using transgenic mice with AMPK tissue conditional expression, that liver-specific activation of this kinase reduces lipogenesis in vivo and entirely protects against liver steatosis induced by high-fructose diet [5]. Moreover, several studies have shown how different nutraceuticals, i.e. pterostilbene or paeoniflorin, could reduce lipid accumulation in rats/mice fed high fructose diet by activating AMPK signaling and thus, promoting β-oxidation and inhibiting lipogenesis [28, 29].

Furthermore, based on the closed correlation between excessive fructose intake, insulin resistance, and NAFLD, several studies indicated that metformin, an AMPK activator, might have therapeutic potential on liver steatosis [30]. Moreover, data obtained by increasing number of works suggest how SGLT2 inhibitors are an attractive option for NAFLD pharmacologic management [31]. Interestingly, Mancini et al. have demonstrated that canagliflozin, a SGL2 inhibitor used in patients with type 2 diabetes, activates AMPK in different cell lines and in mouse liver [32, 33]. Therefore, LC, used in associated with anti-diabetes drugs, could represent a beneficial therapeutic option for NAFLD.

Additionally, AMPK is a Ca^2+^-related protein: its activation may be due to calcium oscillations [34]. We speculated that LC could play an important role on calcium signaling and, to verify our hypothesis, we analyzed CaMKII protein level detecting that LC promoted CaMKII expression and activation (Fig. 3). These results reveal that LC may participate in the regulation of calcium signaling pathways confirming our previous data that revealed how LC influences calcium release from intracellular calcium stores in human osteoblasts [26]. Accumulating evidence of the role of this nutraceutical on calcium homeostasis opens up new therapeutic developments in the pathologies characterized by a calcium impairment, including diabetes mellitus and cardiovascular diseases.

Numerous data suggest that chronic exposure to a high-fructose microenvironment could lead to a decrease in the mitochondrial biogenesis and a concomitant increase in mitochondrial oxidative stress production [35] in hepatocytes.

Our data indicated that LC treatment could counteract fructose action on mitochondrial biogenesis enhancing PGC1 α protein content in steatosis HepG2 (Fig. 4b). As known, PGC1 α, the master regulator of mitochondrial biogenesis, is primary regulated by AMPK [36]. Therefore, LC could maintain mitochondrial homeostasis through AMPK activation and subsequently, PGC1 α synthesis as indicated by results obtained using AMPK inhibitor (compound C) (Fig. 4b, c). This action could attenuate environmental stress caused by fructose.

As before mentioned, oxidative stress is critically involved in NAFLD pathogenesis, because caused lipid peroxidation increasing ROS production. In particular, high fructose micro-environmental stimulates ROS formation [4, 6, 7, 35] and this study confirmed the exacerbated ROS production not only in mitochondria, but also in other cytoplasm compartments (Fig. 4c). Our data reveled as LC had an antioxidant effect on steatosis HepG2 cells: ROS production was alleviated in mitochondria and in cytoplasm. Moreover, the protein content of SOD2 and Nrf2, the main factors involved in cellular antioxidant responses, were increased in LCF condition in respect to F (Fig. 5b).

SOD2, an important antioxidant enzyme present in hepatocytes, thwarts oxidative damages. In fact, it was shown that SOD knockout mice presented abnormal lipid metabolism in the liver. High levels of oxidative stress, caused by defects in the antioxidant system as result of concomitant deficiency of SOD in SOD-knockout mice, resulted in hepatic lipid accumulation via altered lipid metabolism [37].

Our data, obtained by western blot analysis (Fig. 5b, c) showed an incremental LC effect on SOD2, which is inhibited by compound C, although the permanence of a very weak signal suggests a LC residual activity through an independent AMPK mechanism. LC increased SOD2 protein concentration compared to both CNT and F, but its effect is not enough to counteract F presence, supporting the hypothesis that other AMPK-independent pathways are at stake. This evidences agrees with the dichotomous SOD2 regulation described in carcinogenic cells, like HepG2 cell line [38] and support our date showing that F seems to act on SOD2 through both AMPK-dependent and -independent manner.

Various studied have shown that fructose downregulated Nrf2 expression and that some natural products ameliorate liver steatosis by reverting this effect [14, 37, 39]. In agreement with these observations, in our experiment, fructose decreased Nrf2 content while LC treatment recovered Nrf2 protein level (Fig. 5b). More importantly, many works have revealed the crucial role of Nrf2 in progression of NAFLD to steatohepatitis: in mice, Nrf2 depletion increased ROS production and consequently, liver fibrosis and inflammation, the principal pathological features of NASH [37].

It is also important to highlight that numerous data have indicated how Nrf2 expression is regulated by AMPK activation [40] and showed by our results obtained using AMPK inhibitor (Fig. 5c). Consequently, our results suggested that LC-promoting AMPK activity enhances a synergic action on mitochondrial homeostasis and oxidative defense machine that could counteract fructose-induced hepatic steatosis.

Interestingly, the results of this work point out on LC anti-oxidant properties. In line with an increasing number of evidence, our results showed LC ability to reduce ROS production and contemporarily to rise the expression of antioxidant enzymes, i.e. SOD, catalase and phospholipid hydroperoxide glutathione peroxidase in an in vitro, in vivo models and humans [41–44]. In this regard, our group established this LC ability in skeletal muscle cells model, cardiomyocytes and osteoblastic cells observing that this anti-oxidant action is strictly correlated with calcium signaling [18–20, 26, 41]. Additional investigations are needed to better clarify LC antioxidant function and mechanisms.

## Conclusion

This experimental study on human hepatocytes was designed to verify the protective role of LC against fructose-induced liver steatosis and to investigate LC molecular mechanisms of action. In this regard, we studied lipid deposition, mitochondrial homeostasis and antioxidant status, thus determining AMPK activity and CaMKII, SOD2 and Nrf2 level.

Taken together, our findings indicated that LC succeeds, through AMPK activation, in attenuating fructose-induced lipid deposition and, by regulating SOD and Nrf2 activity, preserves mitochondrial homeostasis and strengths cellular antioxidant machine in human HepG2 cells.

This evidence highlights the potential protective effect of this nutraceutical compound in NAFLD.
